# Effectiveness of Nonpharmacological Measures on Improving Headache Score, Strength, Pain, and Quality of Life in Cervicogenic Headaches: A Systematic Review

**DOI:** 10.7759/cureus.57361

**Published:** 2024-03-31

**Authors:** Deepali S Patil, Priya Tikhile, Nikita Gangwani

**Affiliations:** 1 Musculoskeletal Physiotherapy, Ravi Nair Physiotherapy College, Datta Meghe Institute of Higher Education & Research, Wardha, IND

**Keywords:** exercises, manual therapy, quality of life, cervicogenic headache, physical therapy, nonpharmacological measures

## Abstract

Cervicogenic headache (CGH) is a common condition affecting a significant portion of the population and is effectively managed through various interventions, including nonpharmacological approaches. Physical therapy plays a crucial role in CGH management, with numerous studies supporting its effectiveness. This systematic review aimed to evaluate the effectiveness of specific nonpharmacological physical therapy interventions for CGH. A comprehensive search was conducted across various databases (PubMed, Medline, PEDro, and Cochrane Library) for randomized controlled trials (RCTs) published between January 2017 and January 2023 investigating the effectiveness of specific nonpharmacological physical therapy interventions for CGH. We employed manual searches to capture potentially missed studies. Independent reviewers screened all studies based on predefined eligibility criteria. Extracted data included methodology, specific interventions, outcome measures (headache score, strength, pain, and quality of life (QOL)), and study conclusions. Eight RCTs were identified as meeting all inclusion criteria and were thus included in the data synthesis. The findings from these trials revealed a diverse range of nonpharmacological physical therapy interventions, including but not limited to manual therapy, exercise therapy, and multimodal approaches. Specifically, the interventions demonstrated significant improvements in headache scores, strength, pain levels, and overall QOL among individuals with CGH. These results underscore the multifaceted benefits of physical therapy in managing CGH and highlight its potential as a comprehensive treatment option. This review identified eight relevant RCTs investigating nonpharmacological interventions for CGH. Despite the promising findings, this review acknowledges several limitations, including the limited sample size and the heterogeneity of interventions across studies. These limitations emphasize the necessity for further research to elucidate optimal intervention strategies and refine treatment protocols. Nevertheless, the comprehensive analysis presented herein reinforces the pivotal role of physical therapy in not only alleviating pain but also enhancing function and improving the QOL for individuals suffering from CGH.

## Introduction and background

Cervicogenic headache (CGH) often presents with a telltale sign: unilateral neck discomfort. This prevalent and persistent headache frequently arises following a neck movement and can be accompanied by restricted neck mobility [1]. It is crucial to distinguish CGH from other common headaches, like tension headaches or migraines. This exercise delves into the specific causes, symptoms, assessment techniques, and treatment options for various CGH subtypes [2]. The vital role of the interprofessional team in identifying, assessing, and managing CGH is crucial [3]. Despite being recognized for over three decades, CGH remains a treatment challenge. Fortunately, a multimodal approach combining medication and nonpharmacological therapies, like exercise, spinal manipulation, and soft tissue therapy, has proven effective [4-6]. Treatment options can also include minimally invasive percutaneous techniques for targeted pain relief, with surgery reserved for severe cases [7]. Spinal manual therapy (SMT) emerges as a promising nonpharmacological approach for CGH. SMT practitioners believe that it can address underlying mechanical dysfunction in the spine, potentially caused by a manipulable lesion (also called a functional spinal lesion or subluxation), through targeted application of pressure and movement [8].

Research indicates that SMT, involving high-velocity, low-amplitude thrusts to the cervical and upper thoracic spine, can lead to a significant reduction in headache frequency and intensity [9]. Moreover, other techniques, such as suboccipital muscle relaxation, ischemic compression, Mulligan's Sustained Natural Apophyseal Glides (SNAG), and vertebral translatory mobilization, have also been explored for their therapeutic benefits in CGH management [10]. All of these methods have been shown to alleviate symptoms associated with CGH. It looks like upper cervical SMT is the most successful of the many different approaches and procedures [11]. Meanwhile, long-term outcomes can be maintained by including SMT in the treatment plan [12]. The restoration of muscle sarcomere length [13] and myofascial release (MFR) is another approach showing promise for CGH treatment. By targeting the soft tissue, MFR may reduce pain and improve functional outcomes by addressing the myofascial elements that contribute to the headache's pathophysiology [14]. MFR is another promising approach for CGH. By restoring the length and integrity of fascial tissue, MFR may alleviate pain by reducing strain on sensitive structures, like blood vessels and nerves in the area. This is particularly relevant for CGH, as joint soreness is a common characteristic [15]. These findings align with prior research suggesting myofascial and upper cervical joint dysfunction as key contributors to CGH, particularly sternocleidomastoid pain [16].

SMT offers not only the potential for long-term benefits but also the possibility of immediate improvement for CGH patients. One such promising technique is the positional release technique (PRT) [17]. PRT involves applying targeted pressure and positioning the patient in a comfortable posture to address related dysfunction. This gentle approach may be particularly appealing to patients seeking immediate pain relief [18]. During PRT for CGH, the practitioner positions the area of pain in a comfortable posture. This facilitates the relaxation of surrounding tissues and potentially reduces trigger point activity, ultimately aiming to shorten the affected muscle [19]. Patients with CGH often exhibit weakness in their deep neck flexor muscles, which are crucial for neck stability. Studies have confirmed that cervical cranial flexion (CCF) exercises, focusing on controlled neck flexion movements, may be more effective in managing chronic neck pain compared to exercises involving resisted neck flexion. This underscores the potential of CCF exercises to improve function and potentially reduce pain in CGH patients [20]. Interestingly, the strengthening program used was based on research involving women with chronic neck pain [21], and it demonstrated effectiveness in both CGH and persistent neck pain [22].

Given the variety of nonpharmacological interventions available, this systematic review aims to critically evaluate the evidence from recent randomized controlled trials (RCTs) to determine the effectiveness of these approaches in improving headache scores, strength, pain, and quality of life in individuals with CGH. By doing so, we hope to provide a clearer understanding of the role these therapies play in CGH rehabilitation and guide clinical practice in the management of this challenging condition.

## Review

Data sources and search engines

A comprehensive search strategy was employed to identify relevant studies. Following the methodology outlined by Dickersin et al., we conducted a systematic search across several databases, including Medline, Embase, PubMed, PEDro, and the Cochrane Library [23]. The search encompassed studies published between January 2017 and January 2023, with a specific focus on RCTs investigating nonpharmacological interventions, particularly physiotherapy approaches, for the management of CGH.

Methodology

A two-phase search strategy was implemented to ensure a comprehensive review of relevant literature. Following the initial search conducted across Medline, Embase, PubMed, PEDro, and the Cochrane Library, titles, abstracts, and keywords were meticulously reviewed [23]. Keywords included "cervicogenic headache," "nonpharmacological treatment," and "physiotherapy" to identify studies published between January 2017 and January 2023. Both investigators independently assessed the retrieved titles and abstracts. Potentially relevant studies with unclear abstracts or those deemed essential based on initial evaluation were further evaluated by acquiring full-text versions. A secondary search strategy aimed to identify any additional RCTs not captured in the initial database search.

Eligibility criteria

Our review included RCTs that investigated nonpharmacological interventions for CGH. The diagnostic criteria for CGH typically include pain localized to the neck and occipital region, exacerbated by neck movements or sustained awkward head positions, accompanied by a restricted range of motion in the cervical spine, and possibly radiating to the head from the neck or shoulder regions. Studies were selected based on relevance to the topic, not on disease severity, participant age, nationality, or gender.

The inclusion criteria for this review article on physiotherapy interventions for CGH encompass RCTs, quasi-experimental studies, and systematic reviews/meta-analyses published in peer-reviewed journals. Studies must focus on human participants diagnosed with CGH using standardized diagnostic criteria and report outcomes related to headache frequency, intensity, disability, neck pain, range of motion, functional status, QOL, and adverse effects. Physiotherapy interventions of interest include spinal manipulation, mobilization, muscle energy techniques, manual therapy, exercise therapy, and myofascial release. Exclusion criteria involve studies with inadequate methodological quality, incomplete data, animal studies, reviews, editorials, commentaries, conference abstracts, and publications in languages other than English. These criteria are designed to ensure the relevance, methodological rigor, and comprehensiveness of the selected studies, thereby contributing to a robust synthesis of evidence on the effectiveness of physiotherapy interventions for CGH.

Types of intervention

The review specifically focused on RCTs that compared the effectiveness of different physical therapy interventions for CGH against control conditions or standard care practices. These physical therapy interventions, as outlined by the World Confederation for Physical Therapy, encompass a wide range of techniques, including electrotherapy, aerobic exercise, strength training, balance exercises, and foundational body awareness exercises. These interventions can be delivered independently or combined with other modalities, with physical therapy serving as the core active element. The review specifically excluded studies that combined physical therapy with complex weight management programs, as isolating the benefits of physical therapy in these cases would be challenging. Similarly, studies with combined interventions like pharmacotherapy, psycho-education, or cognitive-behavioral strategies were not included. Standard care referred to the usual treatment that patients would receive outside the study, such as hospitalization, outpatient care, or home exercise programs. To ensure comparability, only RCTs with similar intervention durations for both the experimental physical therapy and control groups were included. We then categorized the findings based on their impact on key outcomes for CGH patients, including headache scores, strength, pain levels, and QOL.

Outcome measures

Primary Outcomes

Headache Disability Inventory (HDI): This self-reported questionnaire assesses the impact of headaches on a patient's daily activities. Higher scores indicate greater disability due to headaches.

Neck Disability Index (NDI): This self-reported questionnaire measures limitations in daily activities due to neck pain. Higher scores indicate greater disability.

Numerical Pain Rating Scale (NPRS): This scale quantifies pain intensity on a numerical scale, providing a standardized measure of pain severity. Higher scores indicate greater pain intensity.

CGH frequency: This measure records the number of headache episodes experienced by a patient within a specific timeframe.

Secondary Outcomes

Pain intensity and threshold: These measures assess the severity and tolerance for pain, often using pressure algometry or visual analog scales, providing additional information on pain perception and sensitivity.

Flexion rotation test: This test evaluates cervical spine mobility, particularly rotation, which can be impaired in individuals with CGHs.

QoL: This outcome assesses the overall QOL, encompassing physical, mental, and social well-being, and can reflect the broader impact of headache symptoms on daily functioning.

Cervical range of motion (CROM): This measure quantifies the range of motion in the cervical spine, providing information on mobility and potential restrictions.

Global Rating of Change (GROC): This subjective measure allows individuals to rate their perceived change in symptoms or overall condition, providing a patient-centered perspective on treatment effectiveness.

EuroQol 5D: This standardized instrument assesses health-related QOL across five dimensions, namely, mobility, self-care, usual activities, pain/discomfort, and anxiety/depression, providing a comprehensive assessment of overall health status.

Pain pressure threshold (PPT): This measure evaluates pain sensitivity by applying pressure to specific anatomical points, providing insight into hyperalgesia and central sensitization.

Data extraction and quality evaluation

To ensure methodological rigor, two independent reviewers assessed the quality of each included RCT. Disagreements were resolved through discussion, with a third reviewer providing the final judgment if necessary. The established five-point Jadad scale was used to evaluate the completeness and quality of reporting in each RCT, along with a potential for bias. This widely recognized scale focuses on three key internal validity criteria: withdrawals, double-blinding, and randomization quality. Notably, the Jadad scale is the only psychometrically validated tool available for assessing RCT quality. Higher scores (0-5) indicate stricter adherence to methodological best practices. Studies scoring at least three points were considered high quality, while those with less than three points were considered to have weaker methodologies.

Data synthesis and analysis

To ensure the methodological quality of the included RCTs, we employed the PEDro scale [24], a well-established assessment tool specifically designed for physiotherapy research. This scale offers a comprehensive evaluation of research procedures by considering various factors relevant to physical therapy practice. These factors include participant characteristics, sample size, details of the physical therapy interventions, and the reliability and validity of the outcome measures used in the studies. Two independent reviewers assessed each RCT against the 11 criteria of the PEDro scale (excluding the initial eligibility criterion), assigning a "yes" (criterion met) or "no" (criterion not met) for each.

Search strategy

**Figure 1:**
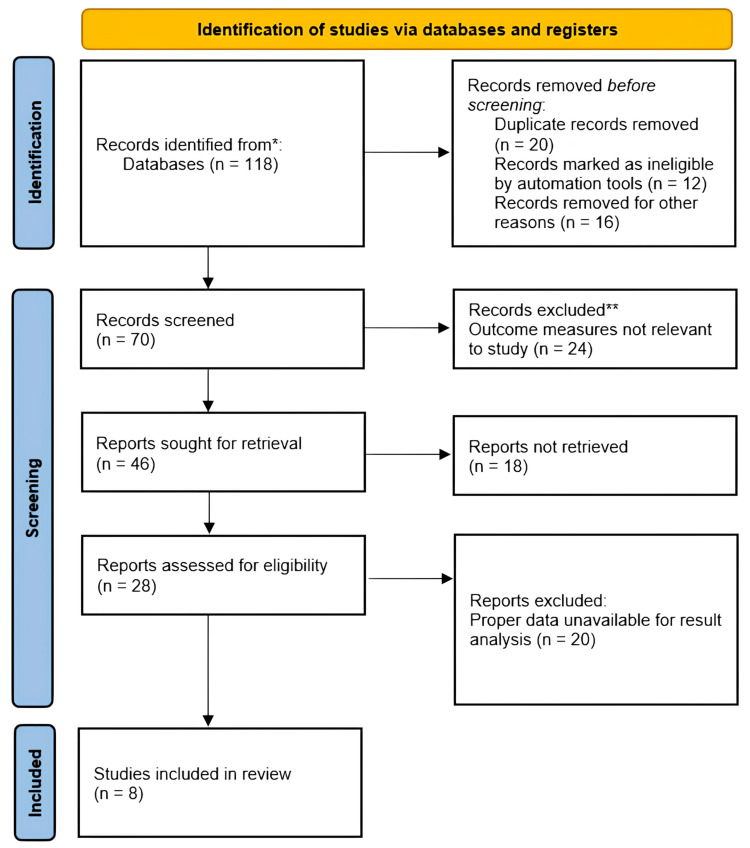
PRISMA flowchart PRISMA: Preferred Reporting Items for Systematic Reviews and Meta-Analyses

Our initial electronic database search yielded 118 relevant articles. To ensure comprehensiveness, we conducted additional manual searches of reference lists, web searches, and consultations with experts. This process identified one additional potentially relevant article. Following duplicate removal and a rigorous screening process involving titles, abstracts, and full texts, a total of eight RCTs were ultimately selected for inclusion in this review. Figure 1 provides a Preferred Reporting Items for Systematic Reviews and Meta-Analyses (PRISMA) flowchart summarizing the article selection process.

A summary of the articles reviewed is mentioned in Table 1.

**Table 1:** Summary of the articles reviewed. TSM: targeted symptom management; CGH: cervicogenic headache; HDI: headache disability inventory; NDI: neck disability index; NPRS: numeric pain rating scale; MMT: mulligan mobilization therapy; SMT: spinal manipulation therapy; CMT: conventional massage therapy; VAS: visual analog scale; NDI: neck disability index; SNAG: sustained natural apophyseal glides; GROC: global rating of change; ACROM: active cervical range of motion; MET: muscle energy technique; IR: infrared radiation; CEHISG: cervicogenic headache international study group; ROM: range of motion; MFR: myofascial release.

Sr. no.	Author and year of publication	Participants	Study setting	Inclusion criteria based on the characteristics of cervicogenic headache	Intervention	Duration of intervention	Outcome measures	Conclusion
1	Annaswamy et al. (2022) [25]	Forty-eight individuals, with an average age of 34.4 years, experiencing symptoms of chronic headache, were included in the study. The participants were randomly assigned to either receive six sessions of TSM or no treatment (Hold). After four weeks, the groups underwent a crossover.	Outpatient rehabilitation	In the study, individuals between the ages of 18 and 65 who reported headaches worsened by neck movements or prolonged postures must have a primary complaint of headache. A physical therapist confirmed the presence of the following criteria to diagnose "probable clinically" CGH: 1) Unilateral or unilateral: A headache that remains consistent on one side, accompanied by neck pain that worsens with certain neck positions or movements [7]. 2) Tenderness in the joints and spreading of symptoms toward the head in at least one of the three upper cervical joints (C0-C3), as determined through weekly manual palpation [9]. 3) Experiencing headaches at least once a week in the past two months.	1. Thoracic spine thrust manipulation. 2. No treatment	One to two times weekly for four weeks, and the treatment session was 15 minutes.	1. HDI, 2. NDI, 3. NPRS	The findings of this research indicate that the implementation of TSM in individuals suffering from chronic CGH did not yield a significant alteration in headache disability, as assessed by the HDI. However, it did result in notable enhancements in pain intensity and neck disability, as evaluated through the NDI.
2	Nambi et al. (2022) [26]	A total of 84 participants from the university hospital were included in the study. These participants were allocated into three groups: the Mulligan mobilization therapy group (MMT; n = 28), the spinal manipulation therapy group (SMT; n = 28), and the control group (Control; n = 28).	Outpatient rehabilitation	1. The pain intensity of CGH ranges from 3 to 8 on a 10-point pain scale. 2. CGH is caused by dysfunction in the cervical spine. 3. There is a decrease in cervical motion. 4. Headache occurs after experiencing neck pain. 5. Patients exhibiting neck stiffness and limited movement were included in the study. Stiffness and movement restrictions were included.	1. MMT, 2. SMT, 3. CMT	Four times a week for four weeks, approximately for 30 minutes. The baseline, four-week, eight-week, and six-month follow-up.	1. CGH frequency. 2. VAS. 3. Headache impact test. 4. Neck pain frequency, pain intensity, and threshold. 5. Flexion rotation test. 6. NDI. 7. Quality of life	Mulligan's mobilization utilizing the SNAG approach yielded superior results in the treatment of cervicogenic headaches compared to both spinal manipulation therapy and conventional massage therapy.
3.	Abdel-Aal et al. (2021) [27]	Total n = 60, with ages 35-50, which were randomly allocated into two groups of 30 participants each.	Outpatient rehabilitation	The condition is commonly observed in individuals aged between 35 and 50 years. The pain is confined to one side of the head and is specifically provoked by external pressure exerted on the upper cervical joints (C1-C3). Furthermore, neck movements can trigger pain, which can persist over a prolonged period. Other contributing factors include uncomfortable positions, limited range of motion in the neck, a minimum pain score of 20 mm on the VAS, experiencing headaches at least once a week for a minimum of three months, and a neck disability index score of 10 points or higher.	1. Graston and exercise group. 2. Exercise group. Three sessions a week for four weeks, approximately 30 minutes.	1. VAS, 2. NDI, 3. cervical range of motion	The immediate impact of incorporating the Graston technique alongside an exercise regimen has been shown to result in a reduction of pain, a decrease in the frequency and duration of headaches, and a lower intake of medication compared to solely engaging in the exercise program over a moderate period.
4.	Young et al. (2021) [28]	The study involved the random allocation of patients into two groups: one group received upper cervical and upper thoracic spinal manipulation along with electrical dry needling (n = 74). In contrast, the other group received upper cervical and upper thoracic spinal mobilization combined with exercise (n = 68).	Outpatient rehabilitation	The headache is exclusively felt on one side and remains localized to that specific area. It originates from the upper back of the neck or the base of the skull, known as the occipital region, and gradually spreads to the forehead, temple, and the area around the eye on the same side. Neck movements or maintaining awkward positions for a prolonged period trigger this pain. In addition, when the head is passively turned to the right or left during a specific test called the flexion-rotation test, it reveals restricted mobility of the neck, measuring less than 32 degrees. The application of pressure to at least one of the upper neck joints, located between the skull and the C3 vertebrae, also induces pain. The headache is described as moderate to severe, but it does not have a pulsating or sharp quality.	Manipulation of the upper cervical and upper thoracic spine involves the skilled application of manual techniques to these specific regions of the spine. This form of treatment aims to restore proper alignment and function, thereby alleviating pain and improving overall musculoskeletal health.	All participants received up to eight treatment sessions at a frequency of once or twice per week over four weeks.	The main measure of interest was the severity of headaches, assessed using the NPRS. Additional measures included the frequency and duration of headaches, level of disability (as determined by the NDI), medication usage, and the GROC.	Patients diagnosed with CGH who underwent thrust spinal manipulation and electrical dry needling exhibited notable enhancements in headache severity, disability, frequency of headaches, duration of headaches, and medication usage in comparison to those who underwent nonthrust spinal mobilization and exercise.
5	Lerner-Lentz et al. (2020) [29]	Forty-five (26 females) patients with cervicogenic headache (mean age 47.8 ± SD 16.9 years) were randomly assigned to receive either pragmatically selected manipulation or mobilization.	Outpatient rehabilitation	To be considered for inclusion in the study, potential patients had to meet specific requirements. These requirements encompassed being within the age range of 18 to 65 years and reporting a primary complaint of headache. Additionally, patients were required to have a confirmed diagnosis of cervicogenic headache, which is characterized by unilateral headache accompanied by neck pain that is exacerbated by specific neck movements or positions. Moreover, patients needed to exhibit tenderness in at least one of the upper cervical joints (C0-C3) as determined through manual palpation. Furthermore, patients had to have experienced a minimum of two headaches in the previous month, possess an NDI score of 20% or higher, and rate their pain intensity as at least 2 out of 10 on the NPRS.	1. Mobilization technique. 2. Manipulation technique.	Not mentioned	1. NDI, 2. NPRS, 3. headache impact test, 4. ACROM	The results of this study demonstrated that there is no significant difference between manipulation and mobilization when applied pragmatically for the management of cervicogenic headaches in terms of pain and disability.
6	Abaspou et al. (2020) [30]	Thirty participants diagnosed with CGH and aged between 18 and 55 years were divided into two groups through a random assignment process. The first group received an intervention consisting of MET applied to the cervical muscles along with IR. On the other hand, the second group served as the control group and only received IR.	Outpatient rehabilitation	CEHISG includes individuals experiencing a persistent unilateral headache, devoid of any shifting between sides, characterized by pain that originates from the occipital region and extends towards the temporal-frontal area for a duration exceeding three months. Additionally, there are reported tenderness and discomfort upon palpation of the upper cervical segments, alongside pain and limitations in movement within the cervical region, particularly evident during upper cervical rotation.	Infrared radiation and muscle energy technique	The interventions will be conducted over a period of six sessions, with each session taking place three times a week for a duration of two weeks.	Headache index, upper cervical rotation ROM, and deep upper cervical muscle thickness	The findings of this investigation may provide substantiation for the advantages of manual therapy in the cervical area for treating CGH. However, it appears that combining MET with IR therapy not only enhances CGH and improves the ROM in the upper cervical region but also yields more long-lasting effects on reducing headache symptoms compared to IR therapy alone.
7	Haas et al. (2018) [31]	The study included 256 adult individuals diagnosed with chronic CGH. The participants were evenly split into two groups and then randomly assigned to receive chiropractic SMT at one of four dosage levels: 0, 6, 12, or 18 sessions.	Outpatient rehabilitation	Participants were selected based on specific criteria aligned with the definition of CGH as outlined by the International Headache Society [4,5]. To be eligible, individuals needed to exhibit additional characteristics indicative of CGH, including a history of experiencing symptoms for a minimum of three months, encountering at least five headaches within the four weeks leading up to the commencement of treatment, sustaining an average pain intensity level of at least 3 on a 0 to 10 pain scale, and demonstrating a clear temporal association linking the origin of their CGH to the neck. Moreover, participants had to meet the criteria for SMT, which involved indications such as cervical joint tenderness or limited motion in cervical joints (either joint play or end play) [23,27]. Furthermore, inclusion criteria encompassed individuals aged 18 or above, capable of walking independently and possessing proficiency in the English language.	1. Spinal manipulation therapy. 2. Light massage therapy.	The participants engaged in the study underwent three sessions per week over a period of six weeks. During the sessions where SMT was not administered, they received a targeted light massage control. Each session lasted a total of 10 minutes.	1. The International Headache Society guidelines for randomized trials recommend headache frequency. 2. Headache Impact test. 3. EuroQol-5D	A persistent correlation was observed between the number of SMT visits and the occurrence of CGH days, which remained consistent for a period of 52 weeks after the initiation of treatment. Notably, the optimal and most impactful dosage of 18 SMT visits resulted in a significant reduction of approximately 50% in CGH days compared to the control group receiving light massage therapy, with an additional average decrease of 3 days per month.
8	Ramezani et al. (2017) [32]	Participants were allocated randomly to a control group focusing on exercise (N = 17, average age = 38 ± 11.31 years) and an experimental group utilizing MFR techniques (N = 17, average age = 38.88 ± 9.38 years).	Outpatient rehabilitation and home-based exercises	The person complains of neck discomfort that extends to the sub-occipital area on one side [6]. Moreover, there's reported pain along with limited movement of the C1-C2 vertebrae during craniocervical flexion, extension, rotation, and tilt [8]. Intensified headaches occur upon manual pressure application to the upper cervical muscles and joints. Additionally, this individual has been suffering from headaches at least once a week consistently for the past six months.	1. Low-load endurance exercises. 2. MFR technique in the upper cervical region.	Each group underwent ten treatment sessions, with a frequency of six times per week.	1. Frequency and duration of headache, intensity of headache. 2. Pressure pain threshold of the spinous and transverse process of upper cervical joints	This study suggests that manual therapy techniques like MET and MFR, combined with exercise, can effectively manage chronic headaches. While MET alone showed potential benefits for cervical headaches, combining it with IR appeared more effective, particularly for improving neck rotation and reducing headache frequency. Furthermore, a correlation was noted between the frequency of SMT sessions and the decrease in headache days, indicating a substantial reduction in symptoms with a higher treatment frequency compared to a control group receiving light massage. These results underscore the effectiveness of manual therapy and exercise in alleviating chronic headaches by enhancing pain management and joint flexibility.

Methodological Quality

The methodological quality of the included RCTs was limited, with a primary concern being the lack of blinding (masking) participants to the intervention they received. Table 2 provides a more detailed breakdown of the studies' characteristics.

**Table 2:** Evaluation of each article's quality using the PEDro scale. PEDro: physiotherapy evidence database; 1: criteria for eligibility; 2: randomized assignment; 3: concealment of allocation; 4: initial measurement of baseline outcomes for each group were similar; 5: participants are kept unaware of the treatment they receive; 6: therapists are blinded to the treatment allocation of individual participants; 7: evaluators are kept unaware of the treatment assignment for each participant; 7: key endpoints are assessed for over 85% of subjects initially randomized; 8: data analysis is carried out following the intention-to-treat principle; 9: group comparisons are presented using statistical analysis; 10: provides both point measures and measures of variability for at least one key outcome.

Sr. No.	Study	1 (not included in the score)	Rating for the criterion										Total score	Main concerns
			2	3	4	5	6	7	8	9	10	11		
1	Annaswamy et al. (2022) [22]	✔	✔	✔	✔	🗶	🗶	🗶	✔	✔	✔	✔	8	No masking (blinding)
2	Nambi et al. (2022) [23]	✔	✔	🗶	✔	✔	🗶	🗶	✔	🗶	✔	✔	7	There is no hidden allocation, blinding, or intention-to-treat analysis in this study.
3	Abdel-Aal et al. (2021) [24]	✔	✔	✔	✔	🗶	🗶	✔	✔	✔	✔	✔	9	No masking of participants and therapist.
4	Young et al. (2021) [25]	✔	✔	✔	✔	🗶	🗶	✔	✔	✔	✔	✔	9	No masking of participants and therapist.
5	Lerner-Lentz et al. (2020) [26]	✔	✔	✔	✔	🗶	🗶	✔	✔	✔	✔	✔	9	No masking of participants and therapist.
6	Abaspour et al. (2020) [27]	✔	✔	✔	🗶	✔	🗶	🗶	✔	✔	✔	✔	8	Baseline outcome measures are not taken. The therapist and assessor are not blind.
7	Haas et al. (2018) [28]	✔	✔	✔	✔	✔	🗶	🗶	✔	🗶	✔	✔	8	The study did not involve blinding of the therapist and assessor. Additionally, there needed to be more intention to treat analysis.
8	Ramezani et al. (2017) [29]	✔	✔	🗶	✔	✔	🗶	🗶	✔	✔	✔	✔	8	No allocation concealment. There was no blinding of the therapist and assessor.

Effectiveness of spinal manipulation on CGH

Nambi et al. investigated the efficacy of spinal manipulation for neck pain and CGH in a three-armed, randomized controlled trial conducted within a hospital physiotherapy department. The study included 237 participants diagnosed with migraine, tension-type headache, or CGH based on standardized criteria. The intervention group received spinal manipulation, specifically mobilization of the thoracic spine, while the control groups received either Mulligan mobilization or traditional massage therapy. It is important to note that in physiotherapy research for CGH, outcome measures are typically assessed at various points throughout the treatment process, including before, after, and at follow-up intervals by an assessor blinded to group allocation. The participants were assigned to one of three groups: exercise alone, exercise plus placebo, or Mulligan mobilization therapy (MMT) plus exercise. Common outcome measures include the Headache Activities of Daily Living Index (HADLI), medication intake, pressure pain threshold (PPT), range of motion assessed by the flexion rotation test, and headache severity and duration. Statistical analyses were conducted following the intention-to-treat principle, using mixed-model analysis of variance (ANOVA) to compare differences between groups at baseline and follow-up assessments, along with 95% confidence intervals. To assess potential differences over time or between groups, additional tests were conducted. A pragmatic study comparing a placebo intervention and exercise to the effectiveness of MMT in reducing headache frequency, intensity, and impairment was carried out. An important limitation to consider was the potential impact of recollection bias on the initial evaluation of headache parameters. The study's external validity was limited to individuals with a minimum one-year history of headaches. There is currently insufficient evidence linking PPT to headache metrics, and the psychometric properties of the HADLI have not been thoroughly investigated. Given that a single therapist administered all treatments, performance bias was inevitable [21].

Building on prior research supporting manual therapy for CGH, this study investigated the comparative effectiveness of different manual therapy techniques. The researchers employed statistical tests (one-way and repeated measures ANOVA) to assess the impact of various interventions on CGH. After four weeks of treatment, the MMT group demonstrated significantly greater improvements (p < 0.001) compared to both the spinal manipulative therapy (SMT) and control groups. These improvements were observed in both primary (frequency of CGH) and secondary outcome measures (CGH pain intensity, disability, neck pain, range of motion, disability index, and QOL). The positive effects of MMT were sustained at the eight-week and six-month follow-up assessments. Improvements in CGH frequency, pain intensity, disability, neck pain, range of motion, function, and QOL were maintained throughout the follow-up period. Interestingly, neck pain tolerance showed a statistically significant increase at the six-month mark compared to the earlier follow-ups (p < 0.05). These findings suggest that MMT, particularly the sustained natural apophyseal glide (SNAG) technique, may be a more effective long-term treatment for CGH compared to spinal manipulation therapy and traditional massage therapy [22].

However, it is important to acknowledge potential sources of heterogeneity among study results, such as variations in participant characteristics, treatment protocols, and outcome measures. In addition, limitations, such as recollection bias and restricted external validity, should be considered when interpreting the findings. Despite these limitations, the study provides valuable insights into the comparative effectiveness of different manual therapy techniques for CGH and underscores the need for further research to elucidate optimal treatment approaches.

Effectiveness of mobilization on CGH

Studies investigated the effectiveness of mobilization techniques for CGH and neck pain. A study focused on Mulligan mobilization, demonstrating its potential benefits for this condition [33]. The study of Dunning et al. used spinal mobilization combined with electrical dry needling and upper cervical and upper thoracic spinal. The common outcome measures used in this study are NDI, NPRS [25], headache impact test [34], and GROC [35]. This study concluded that spinal manipulation and spinal mobilization, along with dry needling, have significant results in the condition of CGH.

Effectiveness of infrared radiation and muscle energy technique on CGH

Solely one investigation carried out by Abaspour et al. analyzed the efficacy of infrared radiation (IR) and muscle energy technique on CGH [23]. The research assessed the headache index, upper cervical rotation range of motion, and deep upper cervical muscle thickness [36]. The results of this study suggested that the amalgamation of muscle energy technique and IR yielded a beneficial effect in alleviating pain among individuals with CGH [30].

Effectiveness of MFR and massage technique on CGH

The researchers in the study have used different types of massage therapy as an intervention for CGH and neck pain [37]. The study by Ramezani et al. combined MFR with low-endurance exercise for neck muscles. The study's outcome measure involves evaluating the frequency and duration of headaches, along with the intensity of these headaches. Furthermore, the research measured the PPT of the spinous and transverse processes of the upper cervical joints [38]. The findings of this study indicated a significant improvement in patients with CGH when a combination of MFR and low-endurance exercise techniques is employed [39], offering a promising outlook for those suffering from these conditions.

Adverse Events

While six studies reported no adverse events associated with massage therapy, two studies documented mild and manageable side effects. Haas et al. noted mild to moderate adverse events, while Dunning et al. observed instances of minor bruising and soreness after dry needling, which were promptly addressed [27,32]. Overall, these findings suggest that massage therapy has a good safety profile, although mild side effects can occasionally occur.

Discussion

CGH presents a significant challenge to patients' QOL [40], and while physical therapy is a cornerstone of management, the evidence base requires strengthening [41]. Our systematic review sought to evaluate the efficacy of various nonpharmacological interventions, identifying eight RCTs that explored techniques, such as spinal manipulation and manual therapy. Despite positive indications, the heterogeneity of techniques precludes a definitive conclusion regarding the most effective approach. A critical limitation was the wide array of physiotherapy techniques, ranging from spinal manipulation to muscle energy techniques, which complicates direct comparisons. For example, while one RCT reported a 30% reduction in headache frequency with spinal manipulation, another found similar improvements with muscle energy techniques, highlighting the difficulty in isolating the effects of individual treatments.

To provide more specific guidance, future research should focus on conducting large-scale RCTs with sufficient statistical power to definitively establish the effectiveness of physiotherapy interventions for CGH. These trials should employ standardized interventions across studies to allow for clearer comparisons between techniques and enhance the generalizability of findings. Furthermore, consistent outcome measures using reliable assessment tools should be utilized to facilitate stronger data analysis and meta-analytic techniques. While positive outcomes were observed, more evidence is desirable to determine the effectiveness of specific physiotherapy interventions for different CGH subtypes. Future studies could investigate the efficacy of various techniques for subgroups defined by factors, like headache chronicity, laterality (pain on the left or right side), or the presence of specific symptoms (e.g., dizziness and nausea).

Regarding the methodological quality of studies, we recognize the importance of addressing potential biases and enhancing the credibility of research in this area. To improve methodological quality, future studies should implement blinding procedures for participants, therapists, and assessors whenever feasible. Blinding helps minimize bias and ensures the reliability of reported outcomes. In addition, researchers should prioritize employing rigorous methodologies and adhering to standardized reporting guidelines to enhance transparency and reproducibility in physiotherapy research for CGH. The methodological quality of some studies could be improved by implementing stricter blinding procedures. Blinding participants, therapists, and outcome assessors can minimize bias and enhance the reliability of findings. In addition, utilizing validated outcome measures with established psychometric properties would strengthen the generalizability of results.

While the review identified positive outcomes from various physiotherapy interventions, it is challenging to pinpoint the single most effective approach due to the heterogeneity of techniques employed across studies. However, based on the available evidence, we can highlight the interventions that consistently demonstrated positive outcomes and may be considered the most promising for CGH management. These interventions include spinal manipulation, mobilization, muscle energy techniques, and other manual therapy approaches. By acknowledging these interventions as the most effective based on the review findings, we aim to provide clear guidance for clinicians and researchers in selecting appropriate physiotherapy modalities for CGH management. Despite limitations, several physiotherapy interventions demonstrated promise across multiple studies. Techniques like spinal manipulation (particularly mobilization techniques) and exercise programs (including deep neck flexor strengthening exercises) showed consistent improvements in headache frequency, intensity, and disability for CGH patients. Future high-quality studies with standardized protocols directly comparing these approaches could provide more definitive evidence about their relative effectiveness. In summary, by addressing the limitations identified and giving specific recommendations for future research, we aim to enhance the credibility and applicability of physiotherapy interventions for CGH management.

## Conclusions

This review aimed to evaluate the effectiveness of physiotherapy interventions for CGH and identify areas for improvement in future research. The main objective was to assess the efficacy of various physiotherapy techniques in managing CGH symptoms. Through a comprehensive analysis of the literature, the review identified several key findings. First, while positive outcomes were observed across different physiotherapy interventions, the lack of standardization in techniques and outcome measures presented challenges in directly comparing their effectiveness and establishing a consistent treatment protocol for CGH. Secondly, variations in outcome measurement tools hindered the ability to perform robust meta-analyses and draw definitive conclusions. Moreover, methodological limitations, such as the lack of blinding and small sample sizes in some studies, may have impacted the reliability and generalizability of the findings. To address these limitations, future research should prioritize implementing standardized interventions, employing consistent outcome measures, and enhancing study methodologies. Moreover, larger-scale trials with sufficient statistical power are necessary to strengthen the evidence supporting physiotherapy interventions for CGH. Moving forward, the establishment of a standardized framework in future research is imperative to facilitate clearer comparisons between interventions and identify the most effective physiotherapy modalities for CGH management. Developing a structured protocol that integrates diverse physiotherapy techniques tailored to individual characteristics could streamline research efforts and enhance the accuracy of treatment recommendations for individuals with CGH.
